# Is Chronic Kidney Disease Affecting the Postoperative Complications of Vitrectomy for Proliferative Diabetic Retinopathy?

**DOI:** 10.3390/jcm10225309

**Published:** 2021-11-15

**Authors:** Yusuke Kameda, Tadashiro Saeki, Ko Hanai, Yuta Suzuki, Yasuko Uchigata, Tetsuya Babazono, Shigehiko Kitano

**Affiliations:** 1 Department of Ophthalmology, Diabetes Center, Tokyo Women’s Medical University School of Medicine, Tokyo 162-8666, Japan; saeki.tadashiro@twmu.ac.jp (T.S.); suzuki.yuta.dmc@twmu.ac.jp (Y.S.); ge2s-ktn@asahi-net.or.jp (S.K.); 2 Department of Medicine, Diabetes Center, Tokyo Women’s Medical University School of Medicine, Tokyo 162-8666, Japan; hanai.dmc@twmu.ac.jp (K.H.); uchigata.dmc@twmu.ac.jp (Y.U.); babazono.dmc@twmu.ac.jp (T.B.); 3 Tokyo Women’s Medical University Medical Center East, Tokyo 162-0054, Japan

**Keywords:** proliferative diabetic retinopathy, chronic kidney disease, vitrectomy, vitreous hemorrhage, neovascular glaucoma

## Abstract

Chronic kidney disease (CKD) is a well-known risk factor for postoperative complications in several surgical fields. However, although prevalent among diabetic candidates for vitrectomy, the effect of CKD on vitrectomy outcomes remains unclear. This study aimed at clarifying the relationship between CKD and the occurrence of vitrectomy-related complications in patients with proliferative diabetic retinopathy (PDR). The 6-month incidences of vitreous hemorrhage (VH) and neovascular glaucoma (NVG) following vitrectomy for PDR were compared among the following groups: stages 1–2 CKD (60 patients), stages 3–5 CKD (70 patients not on hemodialysis), and hemodialysis (HD; 30 patients). We also determined whether the deterioration of the estimated glomerular filtration rate (eGFR) was associated with post-vitrectomy events. The incidence of VH was significantly higher in the stages 3–5 CKD group (43%) than in the stages 1–2 CKD (10%) and HD (10%) groups. NVG was more common in the stages 3–5 CKD group (17%) than in the stages 1–2 CKD (2%) and HD (0%) groups. The reduced estimated glomerular filtration rate (eGFR) was the only significant variable associated with post-vitrectomy VH and NVG. Patients with PDR and CKD, particularly those with lower eGFR, might be at risk for post-vitrectomy VH and NVG.

## 1. Introduction

Proliferative diabetic retinopathy (PDR), which is characterized by the progression of retinal neovascularization, is the most advanced stage of diabetic eye disease. A common complication of PDR is vitreous hemorrhage (VH), which is a major cause of unexpected and severe visual deterioration in patients with diabetes. Vitrectomy is the first-line therapy for intractable VH that significantly improves the anatomical and functional prognoses [1,2]. This advanced instrument is used worldwide; however, postoperative complications may occur in some cases [1].

Common complications of vitrectomy for VH due to PDR include corneal epithelial defects, cataract formation, elevated intraocular pressure, neovascular glaucoma (NVG), iatrogenic retinal breaks, recurrent VH, and rhegmatogenous retinal detachment [1,3,4]. Among these complications, recurrent VH is the most common postoperative event, and has been reported in 7–63% of patients [1]. Similarly, the incidence of NVG, which is regarded as a terminal severe complication, was also present at a higher rate (9.3–11.8%) than other complications, except for VH [1,4].Considering the relationship between perioperative parameters and post-surgical outcomes in vitrectomy for PDR, accumulated evidence suggests that the risk factors for postoperative complications include younger age, male sex, uncontrolled diabetes, insulin administration, fasting blood glucose, and low serum albumin concentration [4,5,6,7,8,9,10]. However, the association between vitrectomy-related complications and chronic kidney disease (CKD) has never been debated, to the best of our knowledge. In contrast, concerns regarding the post-surgical deterioration of patients with CKD have been raised in several surgical fields, such as cardiac, gastrointestinal, hip, and urinary surgery [11,12,13,14,15]. In clinical settings, diabetic retinopathy is often accompanied by CKD since retinopathy and nephropathy usually progress correspondingly, resulting from long-term diabetic microvasculopathy [16,17,18,19,20,21,22,23]. Similarly, diabetic candidates for vitrectomy expectedly possess comorbid conditions [20,21,22,23]. Moreover, many physicians are concerned that patients with PDR and CKD who are on hemodialysis (HD) and undergo vitrectomy may have a higher risk of post-vitrectomy VH, since HD requires systemic anticoagulation using unfractionated heparin [24]. If the deterioration of renal function is associated with an increased risk of postoperative complications of vitrectomy for PDR, it indicates the need for differences in post-surgical management according to the CKD stage. Therefore, we conducted this observational study to determine if different CKD stages in patients with VH due to PDR who need vitrectomy are associated with the occurrence of post-vitrectomy VH and NVG, both of which are often used to estimate the outcome of vitrectomy as the primary endpoint.

## 2. Materials and Methods

### 2.1. Materials

This was a single-center, retrospective, observational cohort study performed using electronic hospital records. The study procedures complied with the tenets of the Declaration of Helsinki. The Institutional Review Board of Tokyo Women’s Medical University approved the study protocol (approval number: 4270, approval date: 25 February 2017) and waived the requirement for informed consent because of the retrospective study design. We included patients with PDR who underwent primary vitrectomy for unresolved VH without tractional retinal detachment at the Department of Ophthalmology, Diabetes Center of Tokyo Women’s Medical University Hospital, in the period from 2011 to 2016. All patients previously received prophylactic laser treatment, mainly panretinal photocoagulation, for PDR before vitrectomy, and all patients had comprehensive ophthalmic examinations daily for 1 week and 1, 2, 3, and 6 months (or more frequently if patients had postoperative events) after vitrectomy. Exclusion criteria were as follows: (1) evidence of vitreoretinal pathology other than PDR, (2) a history of intravitreal administrations of vascular endothelial growth factor (VEGF) inhibitors, (3) the preoperative presence of NVG or tractional retinal detachment associated with PDR, and (4) a surgical procedure combined with gas or silicone tamponade or an injection of VEGF inhibitors. When both eyes met the inclusion criteria, the one that underwent a prior surgery was included. A total of 160 eyes of 160 patients had sufficient baseline and follow-up data for inclusion.

### 2.2. Classification and Outcome Measurement

Patients were stratified into three groups based on kidney function at admission, assessed by the estimated glomerular filtration rate (eGFR), which was estimated using the modified three-variable equation proposed by the Japanese Society of Nephrology: GFR = 194 × age (years)^−0.287^ × serum creatinine level (in mg/dL)^−1.094^ × (0.739 (if female)) [25]. The groups were stages 1–2 CKD (eGFR ≥ 60 mL/min/1.73 m^2^), stages 3–5 CKD (eGFR < 60 mL/min/1.73 m^2^, stages 3a–5 CKD not on HD, and HD (stage 5 CKD on HD). The stages 3–5 CKD had a GFR < 60 mL/min/1.73 m^2^ on at least two occasions 90 days apart according to the definition of CKD.

The primary outcome measure was the 6-month incidence of post-vitrectomy VH and NVG. We compared post-vitrectomy VH and NVG among the stages 1–2 CKD, stages 3–5 CKD, and HD groups. The presence of VH that was not detected at the last fundus examination was considered a VH event during the follow-up period. The severity of postoperative VH was scored on a three-point scale, which was modified from a previous study: grade 1 (mild VH, possible identification of fundus details, hazy view), grade 2 (moderate VH, impaired view of fundus details, optic nerve just visible), and grade 3 (severe VH, no fundus details, optic nerve head invisible) [26].

NVG was defined as the presence of iris/angle neovascularization and elevated intraocular pressure (≥22 mmHg) after vitrectomy. When elevated intraocular pressure was first detected postoperatively, we searched for iris neovascularization using a slit lamp. If neovascularization was absent, gonioscopy was performed.

### 2.3. Risk Factors for Vitreous Hemorrhage or Neovascular Glaucoma

To determine whether renal dysfunction in patients not on HD (i.e., patients in the stages 1–2 CKD and stages 3–5 CKD groups) undergoing vitrectomy was associated with an increased or decreased risk of post-vitrectomy VH and NVG, we divided them into two groups based on the presence/absence of post-vitrectomy VH or NVG within 6 months. We then examined systemic and ophthalmic differences among the groups. The variables at baseline, i.e., perioperatively, that we evaluated in both groups were as follows: age, sex, duration of diabetes, hemoglobin level, hemoglobin A_1c_ level, eGFR, systolic blood pressure, diastolic blood pressure, prescribed medications (insulin therapy and antiplatelet agents), and surgical procedures (combined cataract surgery; endolaser photocoagulation, including the total number of shots; and the removal of fibrovascular proliferation).

### 2.4. Surgical Technique

All patients underwent vitrectomy under local anesthesia. The surgical procedure included a standard 23- or 25-gauge three-port pars plana vitrectomy using the Accurus or Constellation vitrectomy machine (Alcon Laboratories, Inc., Fort Worth, TX, USA). After total vitrectomy with vitreous hemorrhage removal, delamination, endolaser photocoagulation, and endodiathermy were carefully performed to avoid further rebleeding.

In some patients with combined fibrovascular membranes, intraocular forceps were used to peel or remove the epiretinal membranes. During vitrectomy, the vitreous base was thoroughly trimmed, and retinal periphery was inspected to rule out retinal breaks or any other source of bleeding.

When an operated eye had cataract, preventing a clear view of the retina intraoperatively, phacoemulsification and implantation of the round foldable 7.0 mm intraocular lens (Eternity NX-70, Santen Pharmaceutical Co., Ltd., Osaka, Japan) were performed.

### 2.5. Statistical Analyses

Continuous variables are expressed as mean ± standard deviation, and categorical data are expressed as percentage or number. Continuous variables were compared using the unpaired *t*-test or Kruskal–Wallis test, as appropriate. Categorical variables were compared using Fisher’s exact test. Bonferroni post hoc analysis was performed for repeated measurements among the three groups. Differences were considered significant at a two-tailed *p*-value < 0.05. For Bonferroni post hoc analyses, the statistical significance was set at *p* < 0.0167. All statistical analyses were performed using JMP software, version 12.1.0 (SAS Institute Inc., Cary, NC, USA).

## 3. Results

To compare the incidences of post-vitrectomy VH and NVG among the three groups categorized according to kidney function, 60 eyes in the stages 1–2 CKD group, 70 eyes in the stages 3–5 CKD group, and 30 eyes in the HD group were examined. Patient demographics and surgical procedures are summarized in Table 1. Differences among the three groups were not statistically significant, except for the difference in antiplatelet medication (*p* = 0.003, Table 1). In the 6-month period after vitrectomy, VH developed in 6 of 60 (10%) eyes in the stages 1–2 CKD group, 30 of 70 (43%) eyes in the stages 3–5 CKD group, and 3 of 30 (10%) eyes in the HD group, showing a significant difference among the three groups (*p* < 0.001, Table 2), indicating that post-vitrectomy VH events were significantly higher in the stages 3–5 CKD group than in the stages 1–2 CKD group and HD groups (*p* < 0.001 and *p* = 0.002, respectively). Similarly, the 6-month incidence of post-vitrectomy NVG revealed significant overall differences among the three groups (*p* = 0.001, Table 3). Particularly, post-vitrectomy NVG events were significantly higher in the stages 3–5 CKD group (17%, 12/70 eyes) than in the stages 1–2 CKD group (2%, 1/60 eyes) and HD group (0%, 0/30 eyes) (*p* = 0.006 and *p* = 0.016), respectively (Table 3). In post-vitrectomy NVG cases, one eye in the stages 1–2 CKD group and eight eyes in the stages 3–5 CKD group had post-vitrectomy VH events before the onset of NVG. Table 4 and Table 5 show the univariate analysis results of the risk factors for VH and NVG during the 6-month period following vitrectomy in non-HD patients. Both post-vitrectomy VH and NVG groups had lower eGFRs than the event-free group (*p* < 0.001 and *p* = 0.007, respectively); thus, reduced eGFR was the only significant variable associated with post-vitrectomy VH and NVG.

## 4. Discussion

Several clinical studies have shown that PDR is associated with an increased risk for CKD [20,21,22,23]. We realize that patients with diabetes and severe PDR who crucially need vitrectomy are likely to have CKD in real-world settings. However, information about the correlation between CKD and post-vitrectomy complications is lacking. The current study showed that compared to the stages 1–2 CKD and HD groups, patients in the stages 3–5 CKD group were at a higher risk for VH and NVG up to 6 months after vitrectomy for PDR. Therefore, careful postoperative follow-up after vitrectomy for PDR should be performed, particularly in non-HD patients with a significant loss in kidney function.

A few studies have reported that a decline in eGFR increases hemorrhage events [27,28,29,30]. Moreover, previous reports that CKD had a significantly greater risk of postoperative bleeding has been documented in several surgical fields [11,12,15]. Our observational data confirmed these findings, as post-vitrectomy VH was significantly higher in the stages 3–5 CKD group (43%) than in the stages 1–2 CKD (10%) and HD (10%) groups. Regarding post-vitrectomy NVG, Takayama et al. reported that post-vitrectomy VH was a risk factor for the onset of NVG since it might increase VEGF and inflammatory cytokine levels in the aqueous humor, resulting in the development of NVG [4]. In the current study, most (9 of 13 eyes) of the patients with post-vitrectomy NVG had post-vitrectomy VH events before the onset of NVG. Thus, we speculate that the development of post-vitrectomy NVG might be somewhat affected by post-vitrectomy VH.

In contrast, the present study showed that the incidence of post-vitrectomy VH in the HD group was significantly lower compared to the stages 3–5 CKD group, suggesting no association between post-vitrectomy VH and HD. For patients with PDR and CKD on HD, VH seems to be an HD-induced hemorrhagic complication resulting from systemic anticoagulation therapy using unfractionated heparin [24]. However, in our previous study including 145 non-vitrectomized PDR eyes during the 12-month period before and after HD initiation, the incidence of VH was significantly lower in the HD stage (23.4%) than in the pre-HD stage (35.2%), indicating that HD hardly affects VH [31]. In the current study, a similar trend as the previously mentioned findings was observed, even in patients with diabetes and PDR undergoing vitrectomy. Moreover, numerous studies have reported that retinopathy, visual acuity, and macular edema in diabetic eyes stabilized or even improved after HD initiation, although the precise mechanisms are still debated [32,33,34,35,36,37]. In the present study, similar to vitreous hemorrhage, the incidence of post-vitrectomy NVG was not affected by HD because post-vitrectomy NVG developed in the stages 3–5 CKD group (17%) and the HD group (0%), with a statistically significant difference between the groups. Moreover, in some patients in the stages 3–5 CKD group who developed post-vitrectomy NVG, intraocular pressure rapidly decreased to <20 mmHg without surgical intervention immediately after HD initiation and was then kept under control, although it was outside the observation period. Therefore, we hypothesized that the pathogeneses of post-vitrectomy VH and NVG might be suppressed by the stabilizing effect of HD.

Regarding ophthalmic predictors influencing postoperative complications after vitrectomy in patients with diabetes and PDR, numerous recent studies have focused on the relationship between the vitreous level of VEGF and post-surgical outcomes [38,39,40,41,42]. Wakabayashi et al. detected that vitreous VEGF levels during vitrectomy were significantly higher in eyes with early VH and NVG than in those without [38]. Moreover, intravitreal anti-VEGF therapy before or at the end of vitrectomy is considered to prevent post-vitrectomy complications [41,42]. In this retrospective study, we could not measure the vitreous VEGF concentrations during vitrectomy. Therefore, we could not determine whether vitreous VEGF levels are associated with a higher incidence of post-vitrectomy VH and NVG or kidney function. Meanwhile, we eliminated the effect of the anti-VEGF therapy, possibly modifying study outcomes, since we excluded patients who had received such treatment pre- or intra-operatively on the protocol.

The beneficial effects of an intensive multifactorial therapy on renal outcomes are well documented [43,44]. Moreover, Sasso et al. reported that patients with both diabetic kidney disease and severe diabetic retinopathy are likely to have cardiovascular risk, but also that intensive multifactorial therapy for such patients provided a remarkable benefit with regard to this risk [45,46]. In the current study, we could not determine whether this therapy was associated with a degreased risk of post-vitrectomy complications; thus, further investigation is required to clarify this issue.

The present study has some limitations. First, this was a retrospective observational study with a relatively small sample size, particularly of patients in the HD group, possibly causing a selection bias. Second, the retrospective nature of the study could have underestimated the incidence of VH because a small episode of bleeding might have been overlooked, unlike in a prospective study. Finally, patients were followed-up for only 6 months following vitrectomy, and cumulative effects over a longer follow-up period could not be estimated. Within these limitations, the present study suggests that patients with PDR and CKD, particularly those with a lower eGFR, might be at risk for VH and NVG after vitrectomy for PDR. The generalizability of our findings should be confirmed in future prospective studies.

## Figures and Tables

**Table 1:** Clinical characteristics in the three groups.

	Stages 1–2 CKD (*n* = 60)	Stages 3–5 CKD (*n* = 70)	HD (*n* = 30)	*p* Value ^‡^	Significant Difference ^§^
Age, years	58.7 ± 11.5	59.0 ± 10.5	57.2 ± 10.0	0.645	
Male, *n* (%)	39 (65)	55 (79)	26 (87)	0.061	
Duration of diabetes,	17.6 ± 10.5	16.4 ± 10.2	19.5 ± 9.5	0.440	
HbA1c	7.5 ± 1.3	7.4 ± 1.5	7.0 ± 1.3	0.112	
insulin, *n* (%)	34 (57)	40 (57)	17 (57)	1.000	
eGFR	82.7 ± 16.2	35.3 ± 13.1			
Systolic BP	133.7 ± 18.0	136.4 ± 17.9	137.1 ± 19.4	0.600	
Diastolic BP,	75.5 ± 11.7	75.0 ± 11.5	75.1 ± 11.9	0.969	
Antiplatelet agent, *n* (%)	12 (20)	22 (31)	17 (57)	0.003	(I) < (III)
Phacovitrectomy *, *n* (%)	41 (68)	53 (76)	16 (53)	0.084	
PRP shot number ^†^	420 ± 450	330 ± 346	190 ± 211	0.061	
Removal of FM, *n* (%)	05 (8)	09 (13)	04 (13)	0.680	

HbA1c, hemoglobin A_1c_; eGFR, estimated glomerular filtration rate; BP, blood pressure; PRP, panretinal photocoagulation; FM, fibrovascular membrane; CKD, chronic kidney disease; HD, hemodialysis * combined phacoemulsification and vitrectomy; ^†^ intraoperative PRP shot number; ^‡^ statistical analysis of the three groups; ^§^ statistically significant difference between the non-CKD and HD groups using the Bonferroni method.

**Table 2:** Incidence rates of vitreous hemorrhage in the three groups.

	Stages 1–2 CKD (*n* = 60)	Stages 3–5 CKD (*n* = 70)	HD (*n* = 30)	*p* Value ^§^	Significant Difference ^||^
*n* (%)	6 (10)	30 (43)	3 (10)	0.001>	(I) < (II) (III) < (II)
Breakdown					
Grade I *	2	9	2		
Grade II ^†^	1	4	0		
Grade III ^‡^	3	17	1		

* Mild vitreous hemorrhage, possible identification of fundus details, hazy view; ^†^ moderate vitreous hemorrhage, impaired view of fundus details, optic nerve just visible; ^‡^ severe vitreous hemorrhage, no fundus details, optic nerve head invisible; ^§^ statistical analysis of the three groups; ^||^ statically significant difference between two groups using the Bonferroni method.

**Table 3:** Incidence rates of neovascular glaucoma in the three groups.

	Stages 1–2 CKD (*n* = 60)	Stages 3–5 CKD (*n* = 70)	HD (*n* = 30)	*p* Value *	Significant Difference ^†^
*n* (%)	01 (2)	12 (17)	00 (0)	0.001	(I) < (II) (III) < (II)

* Statistical analysis of the three groups; ^†^ statically significant difference between two groups using the Bonferroni method.

**Table 4:** Clinical characteristics of the two non-dialysis groups according to the incidence of vitreous hemorrhage.

	VH (+) (*n* = 36)	VH (−) (*n* = 94)	*p* Value
Age, years	57.2 ± 12.1	59.5 ± 10.4	0.283
Male, *n* (%)	30 (83)	64 (68)	0.124
Duration of diabetes, years	14.7 ± 10.8	17.8 ± 10.1	0.128
HbA_1c_, %	7.2 ± 1.3	7.5 ± 1.4	0.175
insulin therapy, *n* (%)	22 (61)	52 (55)	0.561
eGFR	43.2 ± 22.2	62.5 ± 28.0	0.001> *
Systolic BP, mmHg	135.0 ± 19.1	135.2 ± 17.6	0.966
Diastolic BP, mmHg	75.8 ± 10.8	75.0 ± 11.9	0.753
antiplatelet agent, *n* (%)	07 (19)	27 (29)	0.374
Phacovitrectomy ^†^, *n* (%)	29 (81)	65 (69)	0.273
PRP shot number ^‡^	353 ± 340	379 ± 420	0.744
Removal of FM, *n* (%)	5 (14)	9 (10)	0.531

VH, vitreous hemorrhage; HbA1c, hemoglobin A_1c_; eGFR, estimated glomerular filtration rate; BP, blood pressure; PRP, panretinal photocoagulation; FM, fibrovascular membrane. * Significant *p*-value, ^†^ combined phacoemulsification and vitrectomy; ^‡^ intraoperative PRP shot number.

**Table 5:** Clinical characteristics of the two non-dialysis groups according to the incidence of neovascular glaucoma.

	NVG (+) (*n* = 13)	NVG (−) (*n* = 117)	*p* Value
Age, years	56.9 ± 12.2	59.1 ± 10.8	0.496
Male, *n* (%)	12 (92)	82 (70)	0.111
Duration of diabetes, years	15.2 ± 12.7	17.1 ± 10.1	0.528
HbA_1c_, %	7.1 ± 0.9	7.5 ± 1.4	0.353
insulin therapy, *n* (%)	09 (69)	65 (56)	0.392
eGFR	37.6 ± 16.0	59.4 ± 28.0	0.007 *
Systolic BP, mmHg	139.2 ± 18.8	134.7 ± 17.9	0.388
Diastolic BP, mmHg	78.9 ± 8.1	74.8 ± 11.8	0.226
antiplatelet agent, *n* (%)	05 (38)	29 (25)	0.323
Phacovitrectomy ^†^, *n* (%)	12 (92)	82 (70)	0.111
PRP shot number ^‡^	395 ± 262	369 ± 412	0.823
Removal of FM, *n* (%)	03 (23)	11 (9)	0.148

NVG, neovascular glaucoma; HbA1c, hemoglobin A_1c_; eGFR, estimated glomerular filtration rate; BP, blood pressure; PRP, panretinal photocoagulation; FM, fibrovascular membrane. * Significant *p*-value, ^†^ combined phacoemulsification and vitrectomy; ^‡^ intraoperative PRP shot number.

## Data Availability

All data generated or analyzed during this study are included in this published article.

## References

[1] El Annan J., Carvounis P.E. (2014). Current management of vitreous hemorrhage due to proliferative diabetic retinopathy. Int. Ophthalmol. Clin..

[2] The Diabetic Retinopathy Vitrectomy Study Research Group (1985). Early vitrectomy for severe vitreous hemorrhage in diabetic retinopathy. Two-year results of a randomized trial. Diabetic Retinopathy vitrectomy Study Report 2. The Diabetic Retinopathy Vitrectomy Study Research Group. Arch. Ophthalmol..

[3] Yorston D., Wickham L., Benson S., Bunce C., Sheard R., Charteris D. (2008). Predictive clinical features and outcomes of vitrectomy for proliferative diabetic retinopathy. Br. J. Ophthalmol..

[4] Takayama K., Someya H., Yokoyama H., Takamura Y., Morioka M., Sameshima S., Ueda T., Kitano S., Tashiro M., Sugimoto M. (2019). Risk Factors of neovascular glaucoma after 25-gauge vitrectomy for proliferative diabetic retinopathy with vitreous hemorrhage: A retrospective multicenter study. Sci. Rep..

[5] Motoda S., Shiraki N., Ishihara T., Sakaguchi H., Kabata D., Takahara M., Kimura T., Kozawa J., Imagawa A., Nishida K. (2018). Predictors of postoperative bleeding after vitrectomy for vitreous hemorrhage in patients with diabetic retinopathy. J. Diabetes Investig..

[6] Huang C.H., Hsieh Y.T., Yang C.M. (2017). Vitrectomy for complications of proliferative diabetic retinopathy in young adults: Clinical features and surgical outcomes. Graefe’s Arch. Clin. Exp. Ophthalmol..

[7] Sakamoto M., Hashimoto R., Yoshida I., Ubuka M., Maeno T. (2018). Risk factors for neovascular glaucoma after vitrectomy in eyes with proliferative diabetic retinopathy. Clin. Ophthalmol..

[8] Liao M., Wang X., Yu J., Meng X., Liu Y., Dong X., Li J., Brant R., Huang B., Yan H. (2020). Characteristics and outcomes of vitrectomy for proliferative diabetic retinopathy in young versus senior patients. BMC Ophthalmol..

[9] Sun D.F., Wang Y.L., Wang B., Xu C.L., Zhang G., Li J., Zhang X.M. (2019). Predictive risk factors for exudative retinal detachment after vitrectomy for proliferative diabetic retinopathy. Medicine.

[10] Goto A., Inatani M., Inoue T., Awai-Kasaoka N., Takihara Y., Ito Y., Fukushima M., Tanihara H. (2013). Frequency and risk factors for neovascular glaucoma after vitrectomy in eyes with proliferative diabetic retinopathy. J. Glaucoma.

[11] Acedillo R.R., Shah M., Devereaux P.J., Li L., Iansavichus A.V., Walsh M., Garg A.X. (2013). The risk of perioperative bleeding in patients with chronic kidney disease: A systematic review and meta-analysis. Ann. Surg..

[12] Winkelmayer W.C., Levin R., Avorn J. (2003). Chronic kidney disease as a risk factor for bleeding complications after coronary artery bypass surgery. Am. J. Kidney Dis..

[13] Miyake K., Iwagami M., Ohtake T., Moriya H., Kume N., Murata T., Nishida T., Mochida Y., Isogai N., Ishioka K. (2020). Association of pre-operative chronic kidney disease and acute kidney injury with in-hospital outcomes of emergency colorectal surgery: A cohort study. World J. Emerg. Surg..

[14] You Y., Zhang Y., Qiang L., Sun Y., Zhang J., Bou E., Yan M., Dai K., Ding M. (2019). Prevalence and risk factors for perioperative complications of CKD patients undergoing elective hip surgery. J. Orthop. Surg. Res..

[15] Ning C., Hu X., Liu F., Lin J., Zhang J., Wang Z., Zhu Y. (2019). Post-surgical outcomes of patients with chronic kidney disease and end stage renal disease undergoing radical prostatectomy: 10-year results from the US National Inpatient Sample. BMC Nephrol..

[16] Wong C.W., Wong T.Y., Cheng C.Y., Sabanayagam C. (2014). Kidney and eye diseases: Common risk factors, etiological mechanisms, and pathways. Kidney Int..

[17] Man R.E., Sasongko M.B., Wang J.J., MacIsaac R., Wong T.Y., Sabanayagam C., Lamoureux E.L. (2015). The association of estimated glomerular filtration rate with diabetic retinopathy and macular edema. Investig. Ophthalmol. Vis. Sci..

[18] Grunwald J.E., Alexander J., Ying G.S., Maguire M., Daniel E., Whittock-Martin R., Parker C., McWilliams K., Lo J.C., Go A. (2012). Retinopathy and chronic kidney disease in the Chronic Renal Insufficiency Cohort (CRIC) study. Arch. Ophthalmol..

[19] Park H.C., Lee Y.K., Cho A., Han C.H., Noh J.W., Shin Y.J., Bae S.H., Kim H. (2019). Diabetic retinopathy is a prognostic factor for progression of chronic kidney disease in the patients with type 2 diabetes mellitus. PLoS ONE.

[20] Lin H.T., Zheng C.M., Wu Y.C., Chang Y.H., Chen J.T., Liang C.M., Chang T.J., Zheng J.Q., Tai M.C., Lin Y.F. (2019). Diabetic retinopathy as a risk factor for chronic kidney disease progression: A multicenter case–control study in Taiwan. Nutrients.

[21] Boelter M.C., Gross J.L., Canani L.H., Costa L.A., Lisboa H.R., Três G.S., Lavinsky J., Azevedo M.A. (2006). Proliferative diabetic retinopathy is associated with microalbuminuria in patients with type 2 diabetes. Braz. J. Med. Biol. Res..

[22] Song Y.S., Nagaoka T., Omae T., Yokota H., Takahashi A., Yoshida A. (2016). Systemic risk factors in bilateral proliferative diabetic retinopathy requiring vitrectomy. Retina.

[23] Deva R., Alias M.A., Colville D., Tow F.K.N.F.H., Ooi Q.L., Chew S., Mohamad N., Hutchinson A., Koukouras I., Power D.A. (2011). Vision-threatening retinal abnormalities in chronic kidney disease stages 3 to 5. Clin. J. Am. Soc. Nephrol..

[24] Janssen M.J., van der Meulen J. (1996). The bleeding risk in chronic haemodialysis: Preventive strategies in high-risk patients. Neth. J. Med..

[25] Matsuo S., Imai E., Horio M., Yasuda Y., Tomita K., Nitta K., Yamagata K., Tomino Y., Yokoyama H., Hishida A. (2009). Collaborators developing the Japanese equation for estimated GFR. Revised equations for estimated GFR from serum creatinine in Japan. Am. J. Kidney Dis..

[26] Romano M.R., Gibran S.K., Marticorena J., Wong D., Heimann H. (2009). Can a preoperative bevacizumab injection prevent recurrent postvitrectomy diabetic vitreous haemorrhage?. Eye.

[27] Molnar A.O., Bota S.E., Garg A.X., Harel Z., Lam N., McArthur E., Nesrallah G., Perl J., Sood M.M. (2016). The risk of major hemorrhage with CKD. J. Am. Soc. Nephrol..

[28] Bos M.J., Koudstaal P.J., Hofman A., Breteler M.M. (2007). Decreased glomerular filtration rate is a risk factor for hemorrhagic but not for ischemic stroke: The Rotterdam Study. Stroke.

[29] Holzmann M.J., Aastveit A., Hammar N., Jungner I., Walldius G., Holme I. (2012). Renal dysfunction increases the risk of ischemic and hemorrhagic stroke in the general population. Ann. Med..

[30] Liang C.C., Wang S.M., Kuo H.L., Chang C.T., Liu J.H., Lin H.H., Wang I.K., Yang Y.F., Lu Y.J., Chou C.Y. (2014). Upper gastrointestinal bleeding in patients with CKD. Clin. J. Am. Soc. Nephrol..

[31] Kameda Y., Hanai K., Uchigata Y., Babazono T., Kitano S. (2020). Vitreous hemorrhage in diabetes patients with proliferative diabetic retinopathy undergoing hemodialysis. J. Diabetes Investig..

[32] Berman D.H., Friedman E.A., Lundin A.P. (1992). Aggressive ophthalmological management in diabetic end-stage renal disease: A study of 31 consecutively referred patients. Am. J. Nephrol..

[33] Watanabe Y., Yuzawa Y., Mizumoto D., Tamai H., Itoh Y., Kumon S., Yamazaki C. (1993). Long-term follow-up study of 268 diabetic patients undergoing haemodialysis, with special attention to visual acuity and heterogeneity. Nephrol. Dial. Transplant..

[34] Diskin C.J., Stokes T.J., Dansby L.M., Radcliff L., Carter T.B. (2007). A hypothesis: Can erythropoietin administration affect the severity of retinopathy in diabetic patients with renal failure?. Am. J. Med. Sci..

[35] Yoshimoto M., Matsumoto S. (2006). Changes in diabetic retinopathy and visual acuity in patients with end-stage diabetic nephropathy after the introduction of hemodialysis. Nippon. Ganka Gakkai Zasshi.

[36] Bresnick G.H. (1983). Diabetic maculopathy. A critical review highlighting diffuse macular edema. Ophthalmology.

[37] Perkovich B.T., Meyers S.M. (1988). Systemic factors affecting diabetic macular edema. Am. J. Ophthalmol..

[38] Wakabayashi Y., Usui Y., Okunuki Y., Ueda S., Kimura K., Muramatsu D., Kezuka T., Goto H. (2012). Intraocular VEGF level as a risk factor for postoperative complications after vitrectomy for proliferative diabetic retinopathy. Investig. Opthalmol. Vis. Sci..

[39] Wang J., Chen S., Jiang F., You C., Mao C., Yu J., Han J., Zhang Z., Yan H. (2014). Vitreous and plasma VEGF levels as predictive factors in the progression of proliferative diabetic retinopathy after vitrectomy. PLoS ONE.

[40] Suzuki Y., Suzuki K., Kudo T., Metoki T., Nakazawa M. (2016). Level of vascular endothelial growth factor in the vitreous fluid of proliferative diabetic retinopathy patients and prognosis after vitrectomy. Ophthalmologica.

[41] Liang X., Zhang Y., Wang J.X., Wang L.F., Huang W.R., Tang X. (2019). Intravitreal ranibizumab injection at the end of vitrectomy for diabetic vitreous hemorrhage (Observational Study). Medicine.

[42] Yang C.M., Yeh P.T., Yang C.H., Chen M.S. (2008). Bevacizumab pretreatment and long-acting gas infusion on vitreous clear-up after diabetic vitrectomy. Am. J. Ophthalmol..

[43] Oellgaard J., Gaede P., Rossing P., Persson F., Parving H.H., Pedersen O. (2017). Intensified multifactorial intervention in type 2 diabetics with microalbuminuria leads to long-term renal benefits. Kidney Int..

[44] Tu S.T., Chang S.J., Chen J.F., Tien K.J., Hsiao J.Y., Chen H.C., Hsieh M.C. (2010). Prevention of diabetic nephropathy by tight target control in an asian population with type 2 diabetes mellitus: A 4-year prospective analysis. Arch. Intern. Med..

[45] Sasso F.C., Nicola L.D., Carbonara O., Nasti R., Minutolo R., Salvatore T., Conte G., Torella R. (2006). Cardiovascular risk factors and disease management in type 2 diabetic patients with diabetic nephropathy. Diabetes Care.

[46] Sasso F.C., Pafundi P.C., Simeon V., Nicola L.D., Chiodini P., Galiero R., Rinaldi L., Nevola R., Salvatore T., Sardu C. (2021). Efficacy and durability of multifactorial intervention on mortality and MACEs: A randomized clinical trial in type-2 diabetic kidney disease. Cardiovasc. Diabetol..

